# Odor Fear Conditioning Modifies Piriform Cortex Local Field Potentials Both during Conditioning and during Post-Conditioning Sleep

**DOI:** 10.1371/journal.pone.0018130

**Published:** 2011-03-23

**Authors:** Dylan C. Barnes, Julie Chapuis, Dipesh Chaudhury, Donald A. Wilson

**Affiliations:** 1 Emotional Brain Institute, Nathan Kline Institute for Psychiatric Research, Orangeburg, New York, United States of America; 2 Cognitive Neuroscience Program, City University of New York, New York City, New York, United States of America; 3 Child and Adolescent Psychiatry and Neural Science, New York University School of Medicine, New York City, New York, United States of America; University of Alberta, Canada

## Abstract

**Background:**

Sleep plays an active role in memory consolidation. Sleep structure (REM/Slow wave activity [SWS]) can be modified after learning, and in some cortical circuits, sleep is associated with replay of the learned experience. While the majority of this work has focused on neocortical and hippocampal circuits, the olfactory system may offer unique advantages as a model system for exploring sleep and memory, given the short, non-thalamic pathway from nose to primary olfactory (piriform cortex), and rapid cortex-dependent odor learning.

**Methodology/Principal Findings:**

We examined piriform cortical odor responses using local field potentials (LFPs) from freely behaving Long-Evans hooded rats over the sleep-wake cycle, and the neuronal modifications that occurred within the piriform cortex both during and after odor-fear conditioning. We also recorded LFPs from naïve animals to characterize sleep activity in the piriform cortex and to analyze transient odor-evoked cortical responses during different sleep stages. Naïve rats in their home cages spent 40% of their time in SWS, during which the piriform cortex was significantly hypo-responsive to odor stimulation compared to awake and REM sleep states. Rats trained in the paired odor-shock conditioning paradigm developed enhanced conditioned odor evoked gamma frequency activity in the piriform cortex over the course of training compared to pseudo-conditioned rats. Furthermore, conditioned rats spent significantly more time in SWS immediately post-training both compared to pre-training days and compared to pseudo-conditioned rats. The increase in SWS immediately after training significantly correlated with the duration of odor-evoked freezing the following day.

**Conclusions/Significance:**

The rat piriform cortex is hypo-responsive to odors during SWS which accounts for nearly 40% of each 24 hour period. The duration of slow-wave activity in the piriform cortex is enhanced immediately post-conditioning, and this increase is significantly correlated with subsequent memory performance. Together, these results suggest the piriform cortex may go offline during SWS to facilitate consolidation of learned odors with reduced external interference.

## Introduction

Sleep plays an important role in memory consolidation and its underlying neural plasticity [1], [2], [3], [4], [5], [6]. For example, post-training sleep disruption impairs specific forms of memory ([7], [8] though see [9]), while overnight sleep or even daytime naps [4], [10] improve subsequent memory performance. Indeed, sleep has been linked to emotional, procedural, and declarative memory in both human and non-human animals [11], [12], [13]. Perhaps reflecting the importance of sleep in memory consolidation, sleep structure also can change after training [7], [14], [15]. The changes in sleep related cortical activity can be local, affecting neural activity in specific brain regions especially active during training [16], suggesting an activity-dependent or homeosatic regulation of sleep [17].

Both rapid eye movement sleep (REM) and non-REM or slow-wave sleep (SWS) have been implicated in memory consolidation [2], [17], [18], though they may be differentially involved in declarative and procedural memory [4]. SWS may be particularly important for sleep related memory consolidation. SWS is characterized by slow oscillations (1–5 Hz) of depolarization (up-state) and hyperpolarization (down-state) in widespread thalamic and neocortical neurons [19], [20], and coincident sharp wave-ripples in the hippocampal formation [21], [22]. Neurons in sensory thalamus and neocortex display reduced and/or more variable responses to sensory input during SWS [23], [24], [25], [26] which may reduce interference between external inputs and previously acquired information to be stored [27]. SWS therefore provides a window for neocortical and hippocampal circuits to reactivate pathways and modify synapses involved in specific memory functions [28], [29], [30], [31], [32], [33].

However, it is unclear whether activity in the olfactory cortex shows a relationship between sleep and memory similar to that in thalamocortical systems. The primary olfactory cortex, as opposed to other sensory systems, is not neocortical and has no direct thalamic intermediate between it and the sensory periphery [34]. Despite the lack of a direct thalamic relay however, the olfactory cortex does share some characteristics with thalamocortical sensory systems. For example, the olfactory cortex displays activity shifts between slow-wave and fast-waves states in concert with similar shifts recorded in the neocortex [35], [36]. Furthermore, in anesthetized rats the olfactory cortex becomes less responsive to odors during slow-wave activity compared to fast-wave states [35], [36], and in humans, odors become less arousing during SWS [37], [38]. Importantly, the olfactory cortex, including its largest sub-region the piriform cortex, plays an important role in odor memory, including perceptual learning and associative emotional memory. That is, plasticity within the piriform cortex is critical for perceptual learning and odor discrimination [39], [40], [41]. For example, odor fear conditioning modifies piriform cortical physiology [40], [42], [43], [44], [45], and these cortical changes are associated with enhanced odor perceptual acuity in both humans [40] and rats [46]. Thus, if sleep is important for memory consolidation, learning associated changes in neural activity may be expressed within the olfactory cortex.

The present study had two goals. First, given the diverse effects of anesthesia on olfactory system function [47], [48], we wanted to confirm that odor-evoked activity in the piriform cortex of unanesthetized, chronically recorded rats was reduced during SWS compared to other states. Secondly, we wanted to determine if odor fear conditioning, which modifies olfactory acuity and piriform cortex evoked activity, also modifies sleep structure recorded within the piriform cortex itself during the post-conditioning period. The results suggest that odor fear conditioning modifies piriform cortical responses to the learned odor, and that slow-wave activity, a period of reduced odor responsiveness, is enhanced post-conditioning. This enhanced post-training SWS may facilitate consolidation of the learned odor and its acquired associations.

## Results

### Odor-evoked piriform cortical responses are reduced during SWS

**Figure 1:**
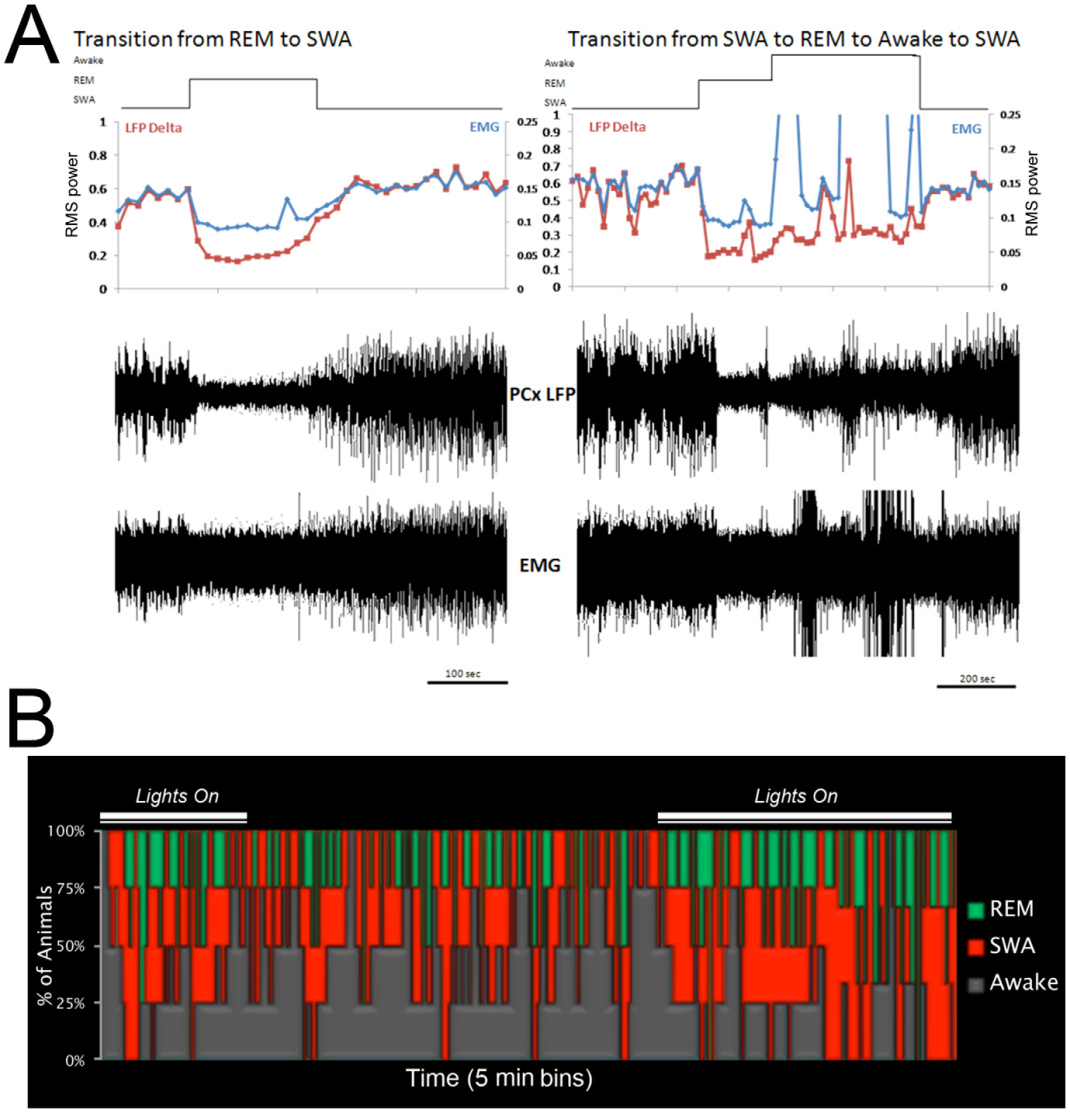
Representative data from one animal showing transition between behavioral states. (**A**) Left, a representative example showing a transition from SWS into REM sleep and returning to SWS as recorded in the anterior piriform cortex (PCX). Each point on the line graph represents a fourteen second time window. The waveforms below are raw LFP and EMG data showing the same window of time as the line graphs above. Note the change in both LFP and EMG frequencies when the animal enters REM sleep. Right, an example of the same animal transitioning from SWS to REM to Awake then returning to SWS. SWS is characterized by high delta power activity and relatively low EMG activity. REM is typified by low delta LFP activity and very low EMG activity. Awake state is distinguished by high frequency activity (lower delta) in the LFP and high frequency EMG waveforms. (**B**) A mean hypnogram recorded in the anterior piriform cortex of 4 naïve rats placed individually in the recording chamber at 3 p.m. for 24 h.

**Figure 2:**
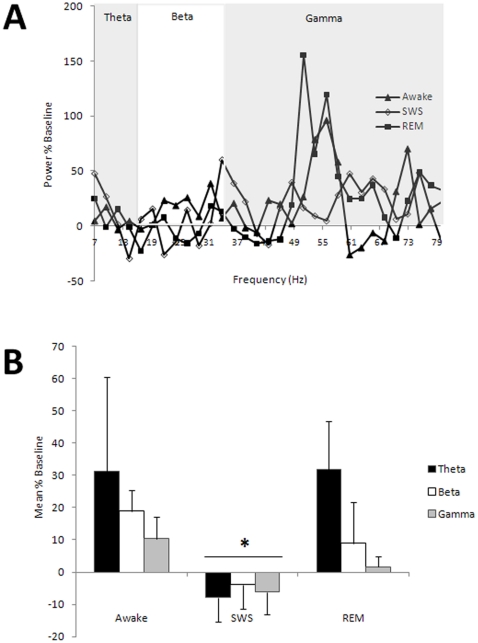
Odor evoked activity in the piriform cortex changes across behavioral states. (**A**) Representative odor evoked anterior piriform cortex activity during awake, REM, and SWS from one animal. During both awake and REM odor stimulation increased activity in the gamma (35–85 Hz) frequency band. There was no obvious odor-evoked activity in the piriform cortex during SWS. (**B**) During Awake and REM, there was significantly greater odor evoked activity in all frequency bands (mean odor-evoked activity ± SEM) compared to SWS.

Based on piriform cortical LFPs and nuchal muscle EMGs, rats (n  =  8) isolated in standard lab cages in a dark, quiet environment for 24 h spent a mean (± S.E.M.) of 13.73±1.35 hrs awake, 9.48±0.99 h in SWS and 2.44±0.24 h in REM. (**Figure 1**). Given previous reports of reduced piriform cortical responsiveness to odors during slow-wave states in urethane anesthetized rats [35], [36], we compared the magnitude of odor-evoked responses during the two fast-wave states (REM and awake periods) and responses during slow-wave sleep in four of these rats. During both awake and REM sleep states, odors evoked a reliable increase in theta (5–15 Hz), beta (15–30 Hz) and gamma (35–85 Hz) frequency activity (**Figure 2B**). On the contrary, odor-stimuli during SWS produced only weak odor-evoked responses in all frequency bands. There was a significant difference in odor-evoked activity between states [F (2,18)  =  4.48, p<0.05]. No odor stimuli examined during SWS occurred within <5 sec of the termination of that SWS period, possibly suggesting that odor stimulation was relatively ineffective in inducing arousal, though this was not systematically examined These data suggest that during an average of 40% of a 24 hour day, the primary olfactory cortex is only weakly responsive to odor input.

### Odor fear conditioning enhances piriform cortical odor-evoked gamma oscillations

**Figure 3:**
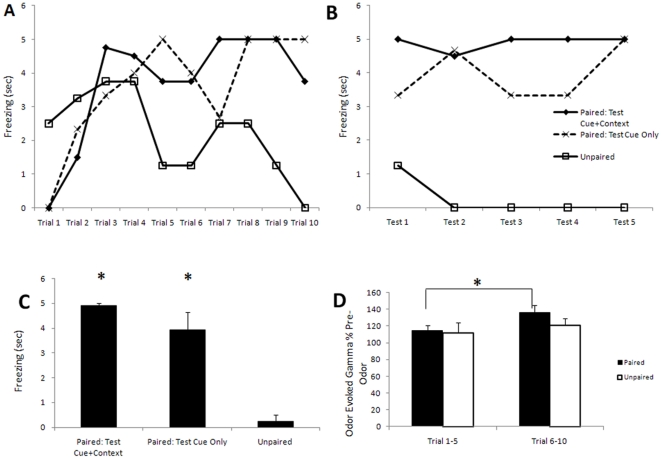
Odor-fear conditioning enhances odor-evoked freezing and odor-evoked gamma oscillations. (**A**) Paired (Test Cue+Context and Test Cue Only) odor-shock animals significantly increased odor-evoked freezing over the course of odor/shock conditioning trials. Furthermore, during odor only tests the following day, (**B**) the Paired animals maintained their odor-evoked freezing response when tested either in the context of the conditioning chamber (n = 6) or in a novel context (n = 4). Unpaired animals (n = 4), however, showed significantly less odor-evoked freezing. During the post-conditioning test, the Unpaired animals show no freezing response to the odor. The legend in Figure B applies also to Figure A. (**C**) Mean level of freezing behavior during the testing session 24 hr post-conditioning across the three groups. (**D**) Power spectrum analysis showed Paired animals had a significant increase in odor-evoked gamma frequency activity over the course of conditioning trials. Odor-evoked gamma (35–85 Hz) activity was significantly higher on average in the second half of trials compared to the first half in Paired animals. There was no change in Unpaired animals. Asterisks signify significant difference between groups.

Paired odor-shock conditioning evoked significantly more odor-evoked freezing behavior during both the conditioning and testing days compared to animals that were conditioned with unpaired stimuli [F (2,112)  = 10.56, p<0.01] (**Figure 3A–B**). Animals that were conditioned with unpaired stimuli did not show acquired odor-evoked freezing during either during training nor during the following testing day. Furthermore, animals that were tested with the cue odor in a different context showed the same odor evoked freezing response as Paired animals tested in the conditioning context. Paired rats tested in the same context and Paired rats tested in a different context showed significantly more odor-evoked freezing than Unpaired rats 24 h post-training [F (2,8)  =  73.38, p<0.001]. Because testing context had no effect on odor memory, the two Paired groups of animals (Cue+Context and Cue Only) were combined for all subsequent analyses.

A power spectrum analysis of LFP recordings in the piriform cortex made during conditioning showed Paired animals had a significant [t (11)  = 2.26, p<0.05] increase in odor evoked gamma frequency activity over the course of trials (**Figure 3C**). More precisely, odor-evoked gamma frequency activity increased during the second half of trials (Trials 6–10) compared to the first half (Trials 1–5) for the Paired animals. There was no increase in odor-evoked gamma activity in Unpaired animals over the course of trials. Furthermore, there was no significant change in theta or beta frequency band activity in either condition. This enhancement in odor-evoked gamma in Paired animals was not maintained on the day of testing. Odor-evoked gamma oscillations were not significantly different between testing and the initial training trials (trials 1–5) in Paired animals nor as compared with odor-evoked gamma on the day of testing in Unpaired animals. There was no significant correlation between the increase in gamma oscillations in Paired animals during training and the post-conditioning SWS duration (r  =  0.22, N.S.).

### Post-conditioning piriform cortical slow-wave activity is enhanced

**Figure 4:**
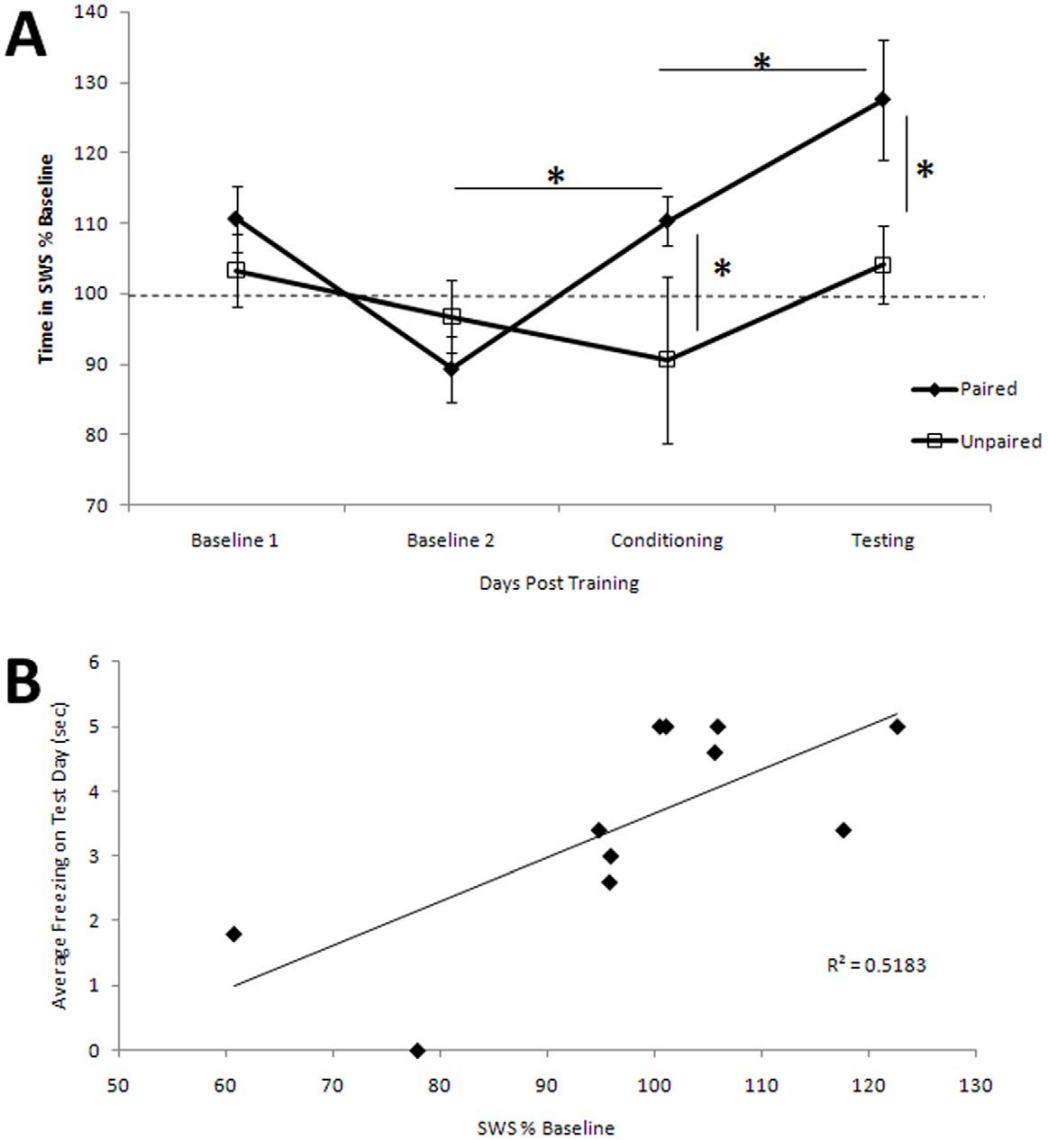
Paired rats increased time spent in post-conditioning SWS. (**A**) Following odor aversion conditioning, paired animals spent more time in SWS recorded in the piriform cortex than on baseline days (mean SWS duration ± SEM). This increase was seen only in odor/shock Paired animals. Immediately following conditioning (marked by Day Conditioning), Paired animals spent significantly more time in SWS than on baseline days and more time than Unpaired animals. This increase was maintained on the day of testing. There was no significant change in REM or total sleep after conditioning in either group (not shown). Asterisks signify significant difference between groups. (**B)** There was a significant correlation between the amount of time spent in SWS immediately after training and the duration of odor-evoked freezing (strength of memory) observed the next day.

Animals that were conditioned with paired odor-shock spent significantly more time in SWS during the 4 h post-conditioning period than they did during pre-conditioning days, and more time than Unpaired animals for the equivalent period of time **(** **Figure 4** **)**. A group X session day ANOVA revealed a main effect of group [F(1,24) = 5.15, p<0.05] and post-hoc Fisher tests revealed a significant difference between Paired and Unpaired time in SWS immediately post-conditioning. A similar difference emerged immediately post-testing (Fisher test, p<0.05). There was also a significant main effect of training day [F(1,3) = 3.35, p<0.05] with post-hoc tests revealing a significant difference between time in SWS post-training compared to baseline days in Paired rats, but not Unpaired rats (p<0.05). Although there was a significant increase in the duration of SWS following conditioning, there was no detectable change in delta oscillation power during the SWS bouts (p>0.05, data not shown).

In contrast to the increase in SWS, there was a non-significant decrease in REM sleep in Paired animals post-training compared to pre-conditioning and Unpaired rats (Paired REM duration post-training as percent of pre-training baseline  =  91.65±19.03; Unpaired  =  159.3±45.46; N.S.). Given the slight decrease in REM and the increase in SWS, there was no significant change in total sleep time after conditioning in either group (Paired total sleep duration post-training as percent of pre-training baseline  =  107.35±5.12; Unpaired  =  100.61±15.52; N.S.).

Finally, we examined if a correlation existed between the amount of change in SWS immediately after training and behavioral performance on the day of testing (odor-evoked freezing duration) in paired animals. Time spent freezing during testing was significantly correlated with the amount of increase in SWS duration during the 4 hr post-training period (r =  0.72, p<0.05), i.e., an increased duration spent in SWS immediately post-training predicted improved memory 24 hr later We also examined if there was a correlation between SWS immediately after training and the cortical response to the conditioned odor (odor-evoked gamma oscillations) on the day of testing in paired animals. There was no significant correlation between odor-evoked gamma oscillations on the day of testing and the duration of SWS immediately after training (r  =  0.23, N.S.).

## Discussion

The results from the present study demonstrate that olfactory fear conditioning modifies neural activity within the piriform cortex both during and after the conditioning session. Odor-evoked LFPs in the piriform cortex during conditioning showed that pairing an odor with foot shock enhanced odor-evoked gamma frequency oscillations over the course of conditioning relative to responses in pseudo-conditioned rats. Furthermore, immediately following conditioning, Paired rats spent significantly more time in SWS compared to pre-conditioning sessions and compared to pseudo-conditioned rats. The amount of time in SWS post-training was significantly correlated with the duration of odor-evoked freezing the following day. There was also an increase in post-testing SWS in Paired rats compared to controls, perhaps reflecting initial extinction effects during these test odor only presentations.

Our results also demonstrate that SWS is associated with reduced piriform responsiveness to odors in unanesthetized animals, which is consistent with sleep-like states in urethane anesthetized rodents [35], [36] and sleep studies in human studies [37], [38]. It is important to emphasize that state-dependent sensory gating appears to occur due to changes within the piriform cortex itself, as only minimal sleep-state dependent changes occur within its primary afferent, the olfactory bulb [35], although specific mechanisms of state-dependent gating need to be further examined with unanesthetized recordings to determine potential contributions of top-down or thalamic modulation not detected in the anesthetized state. Nonetheless, in the unanesthetized rats examined here, odors presented during awake and REM sleep states elicited piriform cortical activity in the theta, beta and gamma ranges while odors presented during SWS evoked significantly less oscillatory activity in all frequency bands. Although this signifies that the piriform cortex is hyporesponsive to odors while in SWS in that odors do not induce robust LFP oscillations, weak odor-evoked activity is still likely to occur [35], [36]. Given that naïve rats spent nearly 40% of the 24 hr day in SWS, this suggests that the piriform cortex spends substantial time in a state that is hypo-responsive to external odors. Furthermore, following conditioning, additional time is spent in this state. We hypothesize that this hypo-responsive state may facilitate odor memory consolidation [33] by reducing external interference [49] while synaptic activity and plasticity induced by recent odor experiences within intracortical circuits are replayed, similar to other systems [50]. In fact, recent work has demonstrated that single-unit activity during slow-wave sleep-like states in anesthetized rats is shaped by recent odor experience during preceding fast-wave states [36]. While this experience-dependent change in activity is consistent with odor replay during sleep, additional work is ongoing to further explore this possibility.

Finally, in addition to potential replay of the learned odors, SWS may also facilitate association of odor quality coding with contextual or emotional information in other circuits such as the amygdala and hippocampus. As noted above, the consequences of odor-fear conditioning include both learning specific associative fear responses such as freezing to the conditioned odor, and also changes in odor acuity, i.e., perceptual learning. Olfactory perceptual learning is strongly associated with changes within the piriform cortex itself [40], [51], [52], while associative and contextual fear conditioning may involve linking piriform cortical activity with multimodal and hedonic representations in other circuits [53]. In support of this, recent work suggests that during SWS-like states in urethane anesthetized rats, piriform cortical activity becomes strongly coherent with activity in the dorsal hippocampus and amygdala, and less coherent with the olfactory bulb [54]. In addition, neocortical up-states during SWS are associated with hippocampal sharp wave-ripples [21], [22], and hippocampal sharp wave-ripples are increased in number and amplitude after odor-reward learning [55]. Thus, when piriform cortical activity becomes less responsive to external odor input during SWS, it becomes more strongly linked to other limbic regions potentially facilitating information transfer and/or neural plasticity between these regions important for associative memory.

In summary, SWS, a period of odor hypo-responsiveness, is enhanced in the piriform cortex following odor fear conditioning. This enhanced SWS may contribute to and/or facilitate odor memory consolidation leading to learned changes in perceptual acuity and changes in learned fear behavior to the conditioned odor. These results suggest that the piriform cortex may function like neocortical systems despite neither having a thalamic input nor having a neocortical architecture [4], [31], [56]. Thus, the role of sleep in memory may be a generalized phenomenon, somewhat independent of specific circuit structure.

## Material and Methods

### Ethics Statement

All experiments were conducted in accordance with the guidelines of the National Institutes of Health and were approved by the Institutional Animal Care and Use Committee of the Nathan Kline Institute, protocol number #AP2009-335.

### Subject

A total of 22 (14 fear conditioning, 8 long-term recordings) male Long-Evans hooded rats (250–450 g) were used as subjects. Animals were housed individually in polypropylene cages on a 12 h light/dark cycle, with food and water available *ad libitum*.

### Electrodes, surgery and histology

**Figure 5:**
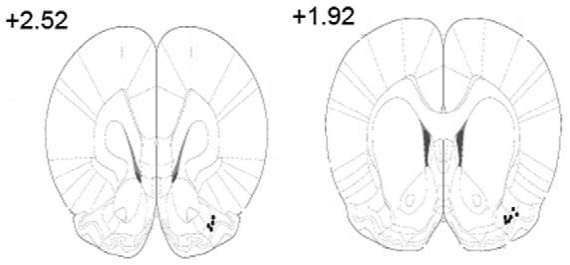
Recording electrode tip locations. Coronal sections of rat's brain with distances from Bregma indicated. The black dots represent the recording site of each piriform cortex LFP. Atlas plates from Paxinos and Watson [58].

Local field potential (LFP) recordings were obtained using Teflon coated 0.18 mm diameter stainless steel electrodes chronically implanted in the anterior piriform cortex. Bilateral electrodes were also implanted in the nuchal muscles to record EMG in all animals except for some rats used for 24 h recordings (see below). All electrodes were connected to a subdermal telemetry pack that was implanted above the animal's left shoulder. Naïve animals were surgically anesthetized with isoflurane throughout the surgical process. An electrode was implanted and cemented on the rat's skull, with the tip in the anterior piriform cortex (1.0 mm anterior to the bregma, 4.5 mm laterally, and 6 mm ventral to the surface of the brain). Antibiotics and analgesics were injected in the rats immediately after the surgery. Animals were given one week for recovery. Following the final recording, rats were given an overdose of urethane and then perfused intracardially with 0.9% saline followed by 10% formaldehyde. Brains removed from the skulls were stored in a 30% sucrose/10% formalin solution for later sectioning. The brains were sectioned coronally at 40 µm, mounted on slides, and stained with cresyl violet. Electrode tracks and locations were verified under a light microscope and marked on a standard brain atlas plate (**Figure 5**).

### Data acquisition, analyses and odor shock conditioning

One week after surgery, recording and training were begun. A standard session included an initial 30 min period alone in a sound and light attenuated recording chamber (30×14×18 cm) to record spontaneous piriform cortex LFPs. The animal was then moved to a stainless steel and Plexiglas conditioning box (30×22×19 cm) with a shock grid floor for 30 min, and then finally placed back in the recording chamber for 4 hours of spontaneous LFP recording. The first several days served as familiarization and baseline sessions and no conditioning occurred. On the day of training animals were divided into 3 groups for the 30 min conditioning session. The Paired group received ten odor-shock pairings with 2 min inter-trial intervals. Each paired odor-shock trial consisted of a five second odor pulse followed immediately by a 1 second, 1 mA foot shock. (+)-Carvone (obtained from Sigma-Aldrich) was used as the odor stimulus. The odor was delivered from a computer controlled flow dilution olfactometer through a port into the conditioning chamber. The odor dissipated from the chamber between trials. The Unpaired control group received 10 shocks (1.5 min inter-trial interval) followed by 10 odor presentations (5 s odor stimulus with 1.5 min inter-trial interval). The third group was trained the same as the Paired group but was tested in a context different than the training chamber. After each daily 30 min session in the conditioning box, the animal was returned to the dark sound attenuated chamber and we recorded LFPs and EMG for 4 hours. Following recording, the animals were then returned to their home cages until the next testing day. The day following training, the rats were again placed in the conditioning chamber or placed in a different context (cue test only: clear polypropylene chamber 40×22×20 cm) and given 5 conditioned odor pulses at 2 min inter-stimulus interval. The rats then again went into the recording chamber for 4 hours. On the conditioning and test days, behavioral (freezing) and LFP responses to the conditioned odor were monitored and recorded. Behavior was videotaped during both the training and testing sessions. Freezing was defined as a cessation of all movement except sniffing, generally with a crouched or arched back posture.

#### Data analysis

LFP and EMG data were collected and analyzed off-line using Spike 2 (CED, Inc.). Fast Fourier Transform (FFT) power analyses were done on the raw LFP data in 14 s intervals to obtain measures of power in 2.4 Hz frequency bins from 0–100 Hz. Power in both the delta (0–5 Hz) and theta (5–10 Hz) frequency bands were calculated for each fourteen second window. To qualify as SWS, an individual 14 s time period had to have an LFP delta value that was higher than the overall delta value for the whole time series and a theta/delta ratio that was less than 0.9 [57]. To calculate REM sleep, we high-pass filtered (100 Hz) activity from the EMG data and low-pass filtered LFP data to remove all high frequency activity (above 5 Hz) to obtain delta frequency activity. To qualify as a REM epoch, both delta and EMG values had to drop below the overall average for delta and EMG respectively [57]. The number of 14 s epochs that met these requirements was then tallied to ascertain the total time spent in REM sleep. We summed REM and SWS sleep totals to obtain the total time spent in sleep during each 4 hour session. To compare the time spent in each stage of sleep across training days, we averaged the total time spent in each stage for the two days preceding the training day and then expressed all values as a percentage of that baseline.

#### Odor-evoked responses

Odor-evoked LFP data were collected during the conditioning and test sessions and analyzed off-line using Spike 2. We performed power spectrum analysis on LFP data and compared the power spectra of five second pre-odor baseline periods to the five seconds of odor presentation in theta (5–10 Hz), beta (15–30 Hz), and high gamma (60–90 Hz) frequency bands.

### Long term recordings

Eight rats were implanted as described above but were not trained in the odor/shock paradigm. Instead, these rats were placed in their home cages inside the recording chamber for 24 hour periods on a 12 h light/dark cycle. LFP recordings were obtained for the entire 24 h period and analyzed in the same method as the odor shock conditioning animals. Two different odors (Carvone and Eugenol obtained from Sigma-Aldrich, 2 s duration) were presented randomly twelve times each over the course of the 24 h period, resulting in some stimuli delivered during fast-wave states and some during slow-wave states. We performed power spectrum analysis on the LFP data and compared odor-evoked activity in delta, theta, beta, and gamma (35–85 Hz) frequency bands, with 5–8 different presentations during each of the different states. Since these random odor presentations did not produce sufficient odor stimuli during REM sleep for analysis, four additional rats were implanted with piriform cortical LFP electrodes and EMG electrodes and continuously monitored for state to allow manual delivery of odors during awake, REM or SWS states.

## References

[1] Stickgold R, Walker MP (2007). Sleep-dependent memory consolidation and reconsolidation.. Sleep Med.

[2] McCarley RW (2007). Neurobiology of REM and NREM sleep.. Sleep Med.

[3] Rauchs G, Desgranges B, Foret J, Eustache F (2005). The relationships between memory systems and sleep stages.. J Sleep Res.

[4] Diekelmann S, Wilhelm I, Born J (2009). The whats and whens of sleep-dependent memory consolidation.. Sleep Med Rev.

[5] Walker MP (2009). The role of slow wave sleep in memory processing.. J Clin Sleep Med.

[6] Deregnaucourt S, Mitra PP, Feher O, Pytte C, Tchernichovski O (2005). How sleep affects the developmental learning of bird song.. Nature.

[7] Leconte P, Hennevin E, Bloch V (1974). Duration of paradoxical sleep necessary for the acquisition of conditioned avoidance in the rat.. Physiol Behav.

[8] Landsness EC, Crupi D, Hulse BK, Peterson MJ, Huber R (2009). Sleep-dependent improvement in visuomotor learning: a causal role for slow waves.. Sleep.

[9] Cai DJ, Shuman T, Harrison EM, Sage JR, Anagnostaras SG (2009). Sleep deprivation and Pavlovian fear conditioning.. Learn Mem.

[10] Tucker MA, Hirota Y, Wamsley EJ, Lau H, Chaklader A (2006). A daytime nap containing solely non-REM sleep enhances declarative but not procedural memory.. Neurobiol Learn Mem.

[11] Cai DJ, Shuman T, Gorman MR, Sage JR, Anagnostaras SG (2009). Sleep Selectively Enhances Hippocampus-Dependent Memory in Mice.. Behav Neurosci.

[12] Gais S, Born J (2004). Low acetylcholine during slow-wave sleep is critical for declarative memory consolidation.. Proc Natl Acad Sci U S A.

[13] Karni A, Tanne D, Rubenstein BS, Askenasy JJ, Sagi D (1994). Dependence on REM sleep of overnight improvement of a perceptual skill.. Science.

[14] Hanlon EC, Faraguna U, Vyazovskiy VV, Tononi G, Cirelli C (2009). Effects of skilled training on sleep slow wave activity and cortical gene expression in the rat.. Sleep.

[15] Magloire V, Cattarelli M (2009). Delayed changes of sleep duration after rewarded olfactory discrimination learning in the rat.. Behav Brain Res.

[16] Huber R, Ghilardi MF, Massimini M, Tononi G (2004). Local sleep and learning.. Nature.

[17] Tononi G (2009). Slow wave homeostasis and synaptic plasticity.. J Clin Sleep Med.

[18] Rasch B, Gais S, Born J (2009). Impaired off-line consolidation of motor memories after combined blockade of cholinergic receptors during REM sleep-rich sleep.. Neuropsychopharmacology.

[19] Steriade M (2006). Grouping of brain rhythms in corticothalamic systems.. Neuroscience.

[20] Buzsaki G (2006). Rhythms of the brain..

[21] Buzsaki G (1996). The hippocampo-neocortical dialogue.. Cereb Cortex.

[22] Molle M, Yeshenko O, Marshall L, Sara SJ, Born J (2006). Hippocampal sharp wave-ripples linked to slow oscillations in rat slow-wave sleep.. J Neurophysiol.

[23] Cohen JD, Castro-Alamancos MA (2010). Behavioral state dependency of neural activity and sensory (whisker) responses in superior colliculus.. J Neurophysiol.

[24] Edeline JM, Manunta Y, Hennevin E (2000). Auditory thalamus neurons during sleep: changes in frequency selectivity, threshold, and receptive field size.. J Neurophysiol.

[25] McCormick DA, Bal T (1994). Sensory gating mechanisms of the thalamus.. Curr Opin Neurobiol.

[26] Rosanova M, Timofeev I (2005). Neuronal mechanisms mediating the variability of somatosensory evoked potentials during sleep oscillations in cats.. J Physiol.

[27] Steriade M, Timofeev I, Grenier F (2001). Natural waking and sleep states: a view from inside neocortical neurons.. J Neurophysiol.

[28] Peyrache A, Khamassi M, Benchenane K, Wiener SI, Battaglia FP (2009). Replay of rule-learning related neural patterns in the prefrontal cortex during sleep.. Nat Neurosci.

[29] Louie K, Wilson MA (2001). Temporally structured replay of awake hippocampal ensemble activity during rapid eye movement sleep.. Neuron.

[30] Lee AK, Wilson MA (2002). Memory of sequential experience in the hippocampus during slow wave sleep.. Neuron.

[31] Pavlides C, Winson J (1989). Influences of hippocampal place cell firing in the awake state on the activity of these cells during subsequent sleep episodes.. J Neurosci.

[32] Ji D, Wilson MA (2007). Coordinated memory replay in the visual cortex and hippocampus during sleep.. Nat Neurosci.

[33] Hasselmo ME, McGaughy J (2004). High acetylcholine levels set circuit dynamics for attention and encoding and low acetylcholine levels set dynamics for consolidation.. Prog Brain Res.

[34] Neville KR, Haberly L (2004). Olfactory cortex..

[35] Murakami M, Kashiwadani H, Kirino Y, Mori K (2005). State-dependent sensory gating in olfactory cortex.. Neuron.

[36] Wilson DA (2010). Single-unit activity in piriform cortex during slow-wave state is shaped by recent odor experience.. J Neurosci.

[37] Carskadon MA, Herz RS (2004). Minimal olfactory perception during sleep: why odor alarms will not work for humans.. Sleep.

[38] Stuck BA, Stieber K, Frey S, Freiburg C, Hormann K (2007). Arousal responses to olfactory or trigeminal stimulation during sleep.. Sleep.

[39] Wilson DA, Kadohisa M, Fletcher ML (2006). Cortical contributions to olfaction: plasticity and perception.. Semin Cell Dev Biol.

[40] Li W, Howard JD, Parrish TB, Gottfried JA (2008). Aversive learning enhances perceptual and cortical discrimination of indiscriminable odor cues.. Science.

[41] Kadohisa M, Wilson DA (2006). Separate encoding of identity and similarity of complex familiar odors in piriform cortex.. Proc Natl Acad Sci U S A.

[42] Sevelinges Y, Gervais R, Messaoudi B, Granjon L, Mouly AM (2004). Olfactory fear conditioning induces field potential potentiation in rat olfactory cortex and amygdala.. Learn Mem.

[43] Sevelinges Y, Sullivan RM, Messaoudi B, Mouly AM (2008). Neonatal odor-shock conditioning alters the neural network involved in odor fear learning at adulthood.. Learn Mem.

[44] Hegoburu C, Sevelinges Y, Thevenet M, Gervais R, Parrot S (2009). Differential dynamics of amino acid release in the amygdala and olfactory cortex during odor fear acquisition as revealed with simultaneous high temporal resolution microdialysis.. Learn Mem.

[45] Chen C-F, Wilson DA (2009). Cortical processing of learned aversive odors in awake rats..

[46] Fletcher ML, Wilson DA (2002). Experience modifies olfactory acuity: acetylcholine-dependent learning decreases behavioral generalization between similar odorants.. J Neurosci.

[47] Rinberg D, Koulakov A, Gelperin A (2006). Sparse odor coding in awake behaving mice.. J Neurosci.

[48] Li AA, Gong L, Liu Q, Li X, Xu F (2010). State-dependent coherences between the olfactory bulbs for delta and theta oscillations.. Neurosci Lett.

[49] Rasch B, Buchel C, Gais S, Born J (2007). Odor cues during slow-wave sleep prompt declarative memory consolidation.. Science.

[50] Stickgold R, Hobson JA, Fosse R, Fosse M (2001). Sleep, learning, and dreams: off-line memory reprocessing.. Science.

[51] Wilson DA (2001). Scopolamine enhances generalization between odor representations in rat olfactory cortex.. Learn Mem.

[52] Wilson DA, Stevenson RJ (2003). Olfactory perceptual learning: the critical role of memory in odor discrimination.. Neurosci Biobehav Rev.

[53] Chapuis J, Garcia S, Messaoudi B, Thevenet M, Ferreira G (2009). The way an odor is experienced during aversive conditioning determines the extent of the network recruited during retrieval: a multisite electrophysiological study in rats.. J Neurosci.

[54] Wilson DA, Yan X (2010). Sleep-like states modulate functional connectivity in the rat olfactory system..

[55] Eschenko O, Ramadan W, Molle M, Born J, Sara SJ (2008). Sustained increase in hippocampal sharp-wave ripple activity during slow-wave sleep after learning.. Learn Mem.

[56] Rowland NC, Goldberg JA, Jaeger D (2010). Cortico-cerebellar coherence and causal connectivity during slow-wave activity.. Neuroscience.

[57] Costa-Miserachs D, Portell-Cortes I, Torras-Garcia M, Morgado-Bernal I (2003). Automated sleep staging in rat with a standard spreadsheet.. J Neurosci Methods.

[58] Paxinos G, Watson C (2009). The rat brain in stereotaxic coordinates, Sixth Edition..

